# STAT2/IRF9 directs a prolonged ISGF3-like transcriptional response and antiviral activity in the absence of STAT1

**DOI:** 10.1042/BJ20140644

**Published:** 2015-03-06

**Authors:** Katarzyna Blaszczyk, Adam Olejnik, Hanna Nowicka, Lilla Ozgyin, Yi-Ling Chen, Stefan Chmielewski, Kaja Kostyrko, Joanna Wesoly, Balint Laszlo Balint, Chien-Kuo Lee, Hans A.R. Bluyssen

**Affiliations:** *Department of Human Molecular Genetics, Institute of Molecular Biology and Biotechnology, Faculty of Biology, Adam Mickiewicz University, Poznan, Poland; †University of Debrecen/Medical Faculty, Department of Biochemistry and Molecular Biology Centre for Clinical Genomics and Personalized Medicine, Debrecen, Hungary; ‡Graduate Institute of Immunology, National Taiwan University College of Medicine, Taipei, Taiwan; §Laboratory of High Throughput Technologies, Institute of Molecular Biology and Biotechnology, Faculty of Biology, Adam Mickiewicz University, Poznan, Poland

**Keywords:** alternative interferon response pathway, cytokines/interferon, host–pathogen interactions, microarray, STAT transcription factor, signal transduction, CCL8, chemokine (C–C motif) ligand 8, CX3CL1, chemokine (C–X3–C motif) ligand 1, Ddx60, DEAD (Asp-Glu-Ala-Asp) box polypeptide 60, DUOX2, dual oxidase 2, EMCV, encephalomyocarditis virus, HA, haemagglutinin, HDACi, histone deacetylase inhibitor, HERC5, HECT and RLD domain-containing E3 ubiquitin protein ligase 5, hST2-U3C, U3C stably overexpressing human STAT2, Ifit1, interferon-induced protein with tetratricopeptide repeats 1, IFI27, interferon alpha-inducible protein, IFN, interferon, IRF9-U3C, U3C stably overexpressing human IRF9, IRF, interferon regulatory factor, ISG, interferon-stimulated gene, ISGF3, interferon-stimulated gene factor 3, ISRE, IFN-stimulated response element, MEF, murine embryonic fibroblast cells, Migr1-MS1KO, MS1KO stably overexpressing Migr1, Migr1-U3C, U3C stably overexpressing Migr1, MOI, multiplicity of infection, MS1KO, STAT1-deficient murine embryonic fibroblast cells, mSTAT2-MS1KO, MS1KO stably overexpressing mouse STAT2, MX1, myxovirus (influenza virus) resistance 1, interferon-inducible protein, NLS, nuclear localization signal, OAS2, 2′-5′-oligoadenylate synthase 2, PKR, protein kinase, interferon-inducible double-stranded RNA-dependent activator, qPCR, quantitative real-time PCR, qRT-PCR, quantitative reverse transcription–PCR, RIG-G, retinoic acid-induced gene G, RSAD2, radical *S*-adenosylmethionine domain-containing 2, SOCS1, suppressor of cytokine signalling 1, STAT, signal transducer and activator of transcription, ΔmSTAT2-MS1KO, MS1KO stably overexpressing mouse ΔmSTAT2, U3C, TNFα, tumour necrosis factor α, VSV, vesicular stomatitis Indiana virus, WT, wild-type

## Abstract

Evidence is accumulating for the existence of a signal transducer and activator of transcription 2 (STAT2)/interferon regulatory factor 9 (IRF9)-dependent, STAT1-independent interferon alpha (IFNα) signalling pathway. However, no detailed insight exists into the genome-wide transcriptional regulation and the biological implications of STAT2/IRF9-dependent IFNα signalling as compared with interferon-stimulated gene factor 3 (ISGF3). In STAT1-defeicient U3C cells stably overexpressing human STAT2 (hST2-U3C) and STAT1-deficient murine embryonic fibroblast cells stably overexpressing mouse STAT2 (mST2-MS1KO) we observed that the IFNα-induced expression of 2′-5′-oligoadenylate synthase 2 (OAS2) and interferon-induced protein with tetratricopeptide repeats 1 (Ifit1) correlated with the kinetics of STAT2 phosphorylation, and the presence of a STAT2/IRF9 complex requiring STAT2 phosphorylation and the STAT2 transactivation domain. Subsequent microarray analysis of IFNα-treated wild-type (WT) and STAT1 KO cells overexpressing STAT2 extended our observations and identified ∼120 known antiviral ISRE-containing interferon-stimulated genes (ISGs) commonly up-regulated by STAT2/IRF9 and ISGF3. The STAT2/IRF9-directed expression profile of these IFN-stimulated genes (ISGs) was prolonged as compared with the early and transient response mediated by ISGF3. In addition, we identified a group of ‘STAT2/IRF9-specific’ ISGs, whose response to IFNα was ISGF3-independent. Finally, STAT2/IRF9 was able to trigger an antiviral response upon encephalomyocarditis virus (EMCV) and vesicular stomatitis Indiana virus (VSV). Our results further prove that IFNα-activated STAT2/IRF9 induces a prolonged ISGF3-like transcriptome and generates an antiviral response in the absence of STAT1. Moreover, the existence of ‘STAT2/IRF9-specific’ target genes predicts a novel role of STAT2 in IFNα signalling.

## Abstract 2

Collectively our results strongly suggest that the alternative IFNα-mediated, STAT2/IRF9 dependent signaling pathway can induce a prolonged ISGF3-like transcriptome and generate an antiviral response analogous to ISGF3, independent of STAT1. Moreover, the existence of “STAT2/IRF9-specific” target genes predicts a novel role of STAT2 in IFNα signaling.

## INTRODUCTION

Interferons (IFNs) are a subset of cytokines that mediate cellular homoeostatic responses to virus infection. IFNs represent a family of molecules which can be divided into three main sub-families: Type I, Type II and Type III [1,2]. Type I IFNs predominantly consist of IFNα and IFNβ subtypes, Type II consists of the single IFNγ type, while Type III comprises IFNλ1, IFNλ2 and IFNλ3 [3]. All IFN types induce IFN-stimulated gene (ISG) expression by phosphorylating STAT1 and STAT2, members of the signal transducer and activator of transcription (STAT) family, mediated by Janus kinases (JAKs). STAT1 homodimers facilitate transcriptional responses to all types of IFN by directly activating genes containing the IFNγ-activated site (GAS) DNA element [4]. Responses to Type I and Type III IFN also depend on STAT2 and the DNA-binding protein interferon regulatory factor (IRF) 9. They form a heterotrimeric transcription complex with STAT1 termed interferon-stimulated gene factor 3 (ISGF3) that binds to the interferon-stimulated response element (ISRE) in ISG promoters [2,5,6]. In ISGF3, STAT2 contributes a potent transactivation domain but is unable to directly contact DNA, whereas STAT1 stabilizes the complex by providing additional DNA contacts [7].

As a component of ISGF3, it is clear that STAT2 plays an essential role in the transcriptional responses to IFN with a strong dependence on STAT1. Previously, we showed that STAT2 is also capable of forming homodimers when phosphorylated in response to IFNα [7]. These STAT2 homodimers were shown to interact with IRF9 and form the ISGF3-like complex STAT2/IRF9 that activates transcription of ISRE-containing genes in response to IFNα [7]. This provides evidence for the existence of STAT1-independent IFNα signalling pathways. In agreement with this, Hahm et al. showed that viruses (like measles virus and lymphocytic choriomeningitis) evade the immune system through a Type I IFN-mediated STAT2-dependent, but STAT1-independent, mechanism [8]. Additionally, STAT2-dependency, but not that of STAT1, was shown by IRF7 expression during viral infection [9]. On the contrary, IRF9 expression in response to IFNα required both STAT1 and STAT2. Similarly, IFNα induction of the antiviral protein apolipoprotein B mRNA-editing enzyme, catalytic polypeptide-like 3G (A3G) and other ISGs [(protein kinase, interferon-inducible double-stranded RNA-dependent activator (PKR), ISG15 and myxovirus (influenza virus) resistance 1 (MX1)] was STAT1-independent, but STAT2-dependent in mouse liver cells. However, STAT1 signalling was functional and required for IFNγ-induction of A3G in these cells [10]. As was suggested by the authors, a potential mechanism responsible for IFNα-induction of A3G could involve STAT2/IRF9-containing complexes. In line with this, chromatin immunoprecipitation (ChIP) analysis using primers specific to ISRE sites confirmed the association of STAT2 with the promoter of antiviral genes induced in response to Dengue virus in STAT1-deficient mice [11]. Lou et al. [12] and Fink et al. [13] provided additional important proof for the biological significance of STAT2/IRF9 complexes in the transcriptional regulation of retinoic acid-induced gene G (RIG-G) and dual oxidase 2(DUOX2), respectively. Lou et al. showed that the STAT2/IRF9 complex effectively drives transcription of the RIG-G gene in NB4 cells upon signalling cross-talk between retinoic acid and IFNα, in a STAT1-independent manner [12]. On the other hand, it was shown that the late antiviral gene DUOX2 was induced by an autocrine/paracrine pathway specifically triggered in airway epithelial cells by synergistic action of IFNβ and tumour necrosis factor alpha (TNFα), and depending on STAT2/IRF9 but not on STAT1 [13]. Therefore, evidence continues to accumulate that IFNα induction of ISGs and biological outcomes can occur in a STAT2/IRF9-dependent, ISGF3-independent manner [14–16]. However, no detailed insight exists into the genome-wide transcriptional regulation and the biological implications of STAT2/IRF9-dependent IFNα signalling as compared with ISGF3.

Our results further prove that an IFNα-mediated, STAT2/IRF9-dependent signalling pathway can induce a prolonged ISGF3-like transcriptional response and generate an antiviral response analogous to ISGF3 in the absence of STAT1. Moreover, we provide evidence for the existence of ‘STAT2/IRF9-specific’ target genes, uncovering a novel role for STAT2 in IFNα signalling, and providing further evidence that IFNα signalling can occur in a STAT2-dependent, STAT1-independent manner.

## EXPERIMENTAL

### Cell culture and reagents

Human fibrosarcoma 2fTGH [17] and STAT1-deficient U3C [18] cells were gifts from Dr Sandra Pellegrini (Institute Pasteur, Paris, France). U3A cells are the standard model for STAT1-null cells [17], derived from a high-frequency mutagenesis screen. U3C cells were selected from the same screen and belong to the same complementation group as U3A, designated U3 (Dr Sandra Pellegrini, Institute Pasteur, Paris, France: personal communication) [17]. Murine embryonic fibroblast cells (MEF) and STAT1-deficient murine embryonic fibroblast (MS1KO) were described previously [19]. Stable cell lines U3C stably overexpressing human STAT2 (hST2-U3C), U3C stably overexpressing Migr1 (Migr1-U3C) and U3C stably overexpressing human IRF9 (IRF9-U3C) were established in our laboratory by co-transfecting (using the calcium phosphate method [20]), U3C cells with the pcDNA6/TR (blasticidin-resistance) plasmid together with the hSTAT2-3xHA-Migr1, empty Migr1 or hIRF9-Migr1 plasmid, respectively. Then, the cells were put on blasticidin (5 μg/ml) (InvivoGen) selection medium, and specific clones were selected based on GFP fluorescence (derived from Migr1 plasmid). MS1KO cells stably overexpressing mouse STAT2 (mSTAT2-MS1KO) or mouse ΔmSTAT2 (ΔmSTAT2-MS1KO) or Migr1 (Migr1-MS1KO) were established as follows: first the calcium phosphate method was used to transfect HEK (human embryonic kidney)-293T cells with mST2-Migr1 or ΔmSTAT2-Migr1 plasmids, respectively, together with GAG-POL and ENV vectors in ratio 3:1:1. After 48 h supernatant containing retrovirus was collected and used for transduction of MS1KO cells as described before [21]. After an additional 24 h, cells were transfected with pcDNA6/TR plasmid using TurboFect transfection reagent (Fermentas). Next, the cells were put on blasticidin (4 μg/ml) selection medium and GFP positive clones were chosen for further characterization.

All cell lines were cultured in Dulbecco's modified Eagle's medium (DMEM, IITD PAN) supplemented with 10% fetal bovine serum (FBS) (PAA Laboratories) and 1% L-glutamine, penicillin/streptomycin (PAA Laboratories).

The cells were stimulated with or without 200 U/ml of recombinant IFNα (Millipore), human cells with human IFNα–IF007 and mouse cells with mouse IFNα–IF009.

### Plasmids and transfection

Human STAT2-3xHA-Migr1, mouse STAT2-Migr1, human IRF9-Migr1 and mouse ΔmSTAT2-3xHA-Migr1 plasmids were constructed in the following way: the full-length cDNA sequence of IRF9 was cloned into the XhoI and EcoRI restriction sites of the MigR1 plasmid [22]. The STAT2 and STAT2-ΔTAD coding sequences (2769 bp and 2199 bp, respectively) combined with the human influenza virus haemagglutinin (HA) epitope (3xHA, 116 bp) were sequentially cloned into the BglII and EcoRI restriction sites of Migr1. The STAT2-Y690F plasmid was constructed using the QuikChange site-directed mutagenesis kit (Agilent). Human STAT2-3xHA-Migr1 plasmid was used as a template and the following primers were designed to introduce the point mutation: For_hSTAT2_Y690F: CAGGAACGGAGGAAATTCCTGAAA-CACAGGCTC; Rev_hSTAT2_Y690F: GAGCCTGTGTTTCAG-GAATTTCCTCCGTTCCTG.

Two transfection methods were used: calcium phosphate method was used as described before [20], and TurboFect transfection reagent was used according to the manufacturer's descriptions (Fermentas).

### Immunoprecipitation and Western blotting

Total cell lysates were prepared by lysing cells in lysis buffer [300 mM NaCl, 50 mM HEPES (pH 7.6), 1.5 mM MgCl_2_, 10% glycerol, 1% Triton X-100, 10 mM sodium pyrophosphate, 20 mM NaF, 1 mM EGTA, 0.1 mM EDTA, 1 mM DTT, 1 mM PMSF and 1 mM Na_3_VO_4_] at 4°C for 20 min. Lysates were quantified by the BCA method (Thermo Scientific) and equal amounts of samples were resolved by 8% SDS/PAGE, followed by transfer to PVDF membrane (Santa Cruz) and Western blot analysis with indicated antibodies. For immunoprecipitation of HA-tagged human STAT2, total cell lysates were subjected to overnight incubation with 5 μg of anti-HA antibody (05–904; Millipore) and 30 μl of Protein G-Sepharose beads (BioVision). The immunoprecipitates were washed according to the manufacturer's instructions and processed for Western blotting. To control for specificity, we additionally performed IP with an unrelated antibody (IgG) (not shown).

Proteins were immunodetected using α-tubulin (04-1117; Millipore), phosphorylated STAT2 (pSTAT1) (07-224; Millipore), ISGF-3γ p48 (sc-10793; Santa Cruz), human total STAT2 (tSTAT2) (sc-839; Santa Cruz), total STAT1 (tSTAT1) (sc-346; Santa Cruz), phosphorylated STAT1 (pSTAT1) (sc-7988-R; Santa Cruz), mouse total STAT2 (tSTAT2) [23] diluted in TBS-T containing either 0.125% non-fat milk or 1% BSA (BioShop). Next, the horseradish peroxidase (HRP)-conjugated goat anti-rabbit IgG secondary antibody (12-348; Millipore) was applied and immunoreactive bands were visualized by enhanced chemiluminescence using the Luminata Forte HRP Substrate (Millipore) and detected with the G:Box System (Syngene).

### Quantitative reverse transcription-PCR (qRT-PCR) analysis

Total RNA was prepared using the GeneMATRIX purification kit (EURx) following the manufacturer's instructions. Total RNA (500 ng) was subjected to reverse transcription and PCR amplification was performed in Maxima SYBR Green/ROX qRT-PCR Master Mix (Fermentas) on the Eco qRT-PCR System (Illumina). Sequences of oligonucleotides (Genomed) are available from H.A.R.B. on request. The amount of target gene in each sample was normalized to endogenous control ACT-β (ΔCT). Data were transformed as described previously [24].

### Microarray and data analysis

First, human 2fTGH and hST2-U3C and mouse MEF wild-type (WT) and mSTAT2-MS1KO cells were treated with or without IFNα for different times: 0 h, 4 h, 8 h, 24 h. RNA from each sample was isolated and labelled via the Illumina® TotalPrep™ RNA Amplification Kit (Life Technologies). Standard Illumina Expression BeadChip HumanHT-12v4 or MouseRef-8v2 (Illumina) hybridization protocols were used to obtain the raw data. Chips were scanned using the HiScanSQ system (Illumina). The complete data of the Illumina Expression BeadChip analysis is available at NCBI GEO, with the accession number GSE50007. The average gene expression signals from three (for human cells) or two (for the mouse cells) independent biological experiments were taken for statistical testing. Background subtraction and quantile normalization were applied and genes significantly (*p*-value≤0.05) up-regulated at least 2-fold in any of the IFNα-treated samples were selected for further analysis. Statistically significant up-regulated genes in different cell-line data sets were compared by Venn diagram analysis (http://bioinfogp.cnb.csic.es/tools/venny/index.html) [25]. Identification of overlapping genes between human and mouse data sets was based on ‘Gene ID and name’. Cluster analysis was performed using Genesis software (http://genome.tugraz.at/genesisclient/genesisclient_description.shtml) [26]. For hierarchical clustering the average linkage method was applied. Thus, induction ratio of common up-regulated genes between human 2fTGH and hST2-U3C or mouse MEF WT and MST2-MS1KOwas log_2_-transformed and subjected to cluster analysis. The automatic gene cluster assignment method was used to create gene clusters. For the common up-regulated genes listed in Table 2, promoter regions from −450 bp to +50 bp (in relation to the transcriptional start site) were searched for the presence of an ISRE sequence according to the Transfac database (PSCAN software; http://www.beaconlab.it/pscan) [27].

Enrichment in gene ontology (GO) categories was performed using Gorilla software (http://cbl-gorilla.cs.technion.ac.il/) [28]. A *P*-value of 10^−3^ was used as a threshold and Illumina gene lists from HumanHT-12 v4 or MouseRef-8 v2 were taken as a background model. Next, all the statistically significant and enriched GO categories were analyzed by Revigo software (http://revigo.irb.hr/) [29]. To remove redundant GO terms, the allowed similarity value of 0.5 was used.

### Chromatin immunoprecipitation

ChIP was performed as described previously [30] with minor modifications. Briefly, cells were treated with IFNα for 0 h and 24 h, followed by cross-linking with DSG (Sigma) for 30 min and then with formaldehyde (Sigma) for 10 min. After fixation chromatin was sonicated with a Diagenode Bioruptor Plus to generate 200–1000 bp fragments. Chromatin was immunoprecipitated with a pre-immune IgG (Millipore, 12-371B) or a polyclonal antibody against STAT2 (Santa Cruz, sc-476X). Chromatin–antibody complexes were precipitated with anti-IgA and anti-IgG paramagnetic beads (Life Technologies). After six washing steps, complexes were eluted and the cross-links reversed. DNA fragments were column purified (Qiagen, MinElute). DNA was quantified with a Qubit fluorometer (Invitrogen). Immunoprecipitated DNA was quantified by quantitative PCR (qPCR) and normalized to values obtained after amplification of unprecipitated (input) DNA. Sequences of oligonucleotides (Genomed) are available on request.

### Antiviral assay

Antiviral assay was performed as described before [21,31] with modifications. 2fTGH, U3C, hST2-U3C and Migr1-U3C cells were seeded on to 96-well plates at 7 × 10^3^ cells/well. Next day, cells were pretreated with or without 2-fold serial dilutions of IFNα, starting from 250 U/ml for 24 h. Subsequently, encephalomyocarditis virus (EMCV) or VSV (vesicular stomatitis Indiana virus) at a multiplicity of infection (MOI) of 0.3 or 3 was added to the cells using serum-free DMEM. Twenty hours post-infection, the medium was removed and cells were fixed with 10% formaldehyde solution for 20 min at room temperature. After fixation, cells were visualized by crystal violet staining. Excess dye was removed by immersing the plate in water.

## RESULTS

### The abrogated IFNα response in STAT1 KO cells correlates with diminished STAT2 phosphorylation

**Figure 1:**
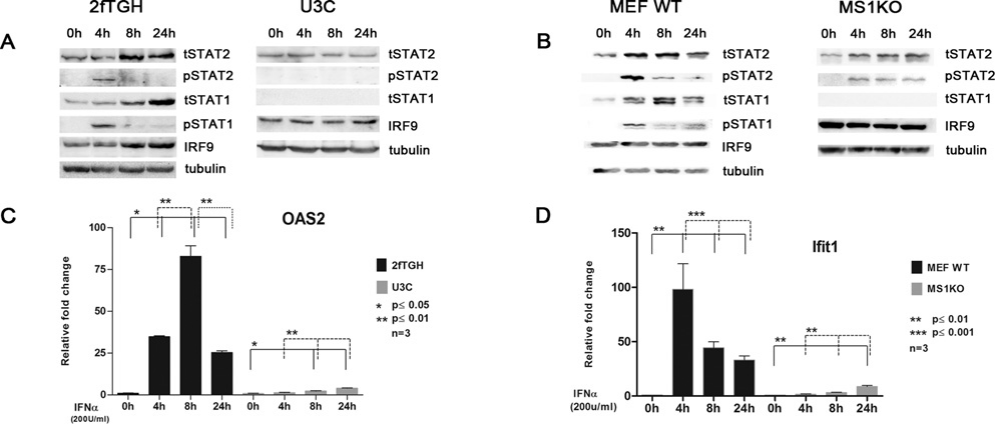
The IFNα response in STAT1 KO cells is abrogated (**A**, **C**) 2fTGH and U3C; (**B**, **D**) MEF WT and MS1KO were treated with IFNα for indicated times. For (**A**) and (**B**), protein lysates were isolated and analyzed by Western blot analysis. Total STAT2 (tSTAT2), phosphorylated STAT2 (pSTAT2), total STAT1 (tSTAT1), phosphorylated STAT1 (pSTAT1) and IRF9 were analyzed using specific antibodies. Equal loading was verified using anti-tubulin. For (**C**) and (**D**), total RNA was extracted and OAS2 and Ifit1 relative fold induction was quantified using qRT-PCR. Statistical significance is presented as compared with the non-treated control (results are means ±S.E.M.). Statistical analysis was conducted using one-way ANOVA with Tukey's *post hoc* test. *P≤0.05, ***P≤0.01.

First, we characterized IFNα responses of the human 2fTGH (WT) and U3C (STAT1-deficient) cell lines, and the mouse MEF (MEF WT) and MS1KO cells. Both human and mouse WT cells were treated with IFNα for increasing times, which resulted in a similar phosphorylation pattern of STAT1 and STAT2. Phosphorylation of both proteins increased after 4 h of treatment and diminished to near basal levels after 8 and 24 h (Figures 1A and 1B). Expression of STAT1 and STAT2 clearly increased in time in 2fTGH and MEF WT in response to IFNα. The expression of IRF9, on the other hand, only increased in 2fTGH. The IFNα response in both U3C and MS1KO cells exhibited diminished STAT2 phosphorylation, despite the normal expression of STAT2 and IRF9 proteins (Figures 1A and 1B). STAT2 phosphorylation in IFNα-treated human U3C cells was not detectable (Figure 1A), even after 1 h and 2 h of treatment (not shown). In mouse MS1KO cells diminished phosphorylation of STAT2 could be detected with more prolonged kinetics as compared with MEF WT cells (Figure 1B). Expression of STAT2 and IRF9 did not increase over time in response to IFNα (Figures 1A and 1B). However, the IFNα-induced expression of the classical ISGs human 2′-5′-oligoadenylate synthase 2 (OAS2) and mouse interferon-induced protein with tetratricopeptide repeats 1 (Ifit1) still slowly increased over time, but at a much lower level as compared with the WT cells (Figures 1C and 1D). Together these results show that the decrease in STAT2 phosphorylation correlated with the diminution of OAS2 and Ifit1 gene expression, suggesting the involvement of STAT2 in IFNα-induction of the latter genes.

### STAT1 KO cells overexpressing STAT2 recapitulate IFNα response

**Figure 2:**
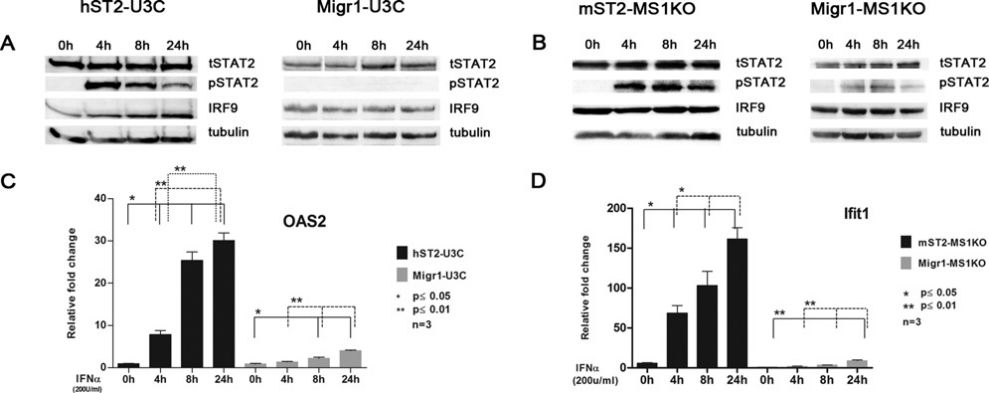
The IFNα response in STAT1 KO cells is recapitulated by increasing STAT2 levels (**A**, **C**) hSTAT2-U3C and Migr1-U3C; (**B**, **D**) mST2-MS1KO and Migr1-MS1KO, were treated with IFNα for the indicated times. For (**A**) and (**B**), protein lysates were isolated and analyzed by Western blot analysis for expression of tSTAT2, pSTAT2, tSTAT1, pSTAT1 and IRF9. Equal loading was verified using anti-tubulin. In (**C**, **D**), total RNA was extracted and OAS2 and Ifit1 relative fold induction was quantified using qRT-PCR. Statistical significance is presented as compared with the non-treated control (results are means±S.E.M.). Statistical analysis was conducted using one-way ANOVA with Tukey's *post hoc* test. *P≤0.05, **P≤0.01.

To study the role of STAT2 and IRF9 in the residual IFNα-induced gene expression in the STAT1 KO cells, we next generated human and mouse STAT1 KO cells overexpressing STAT2 (hST2-U3C and mST2-MS1KO, respectively) or empty vector (Migr1-U3C and Migr1-MS1KO, respectively). IFNα treatment of hST2-U3C and MST2-MS1KO for increasing times resulted in high levels of STAT2 phosphorylation, still being present after 24 h (Figures 2A and 2B). This correlated with the increased expression of IRF9 in hST2-U3C, but not in MST2-MS1KO, cells (Figures 2A and 2B). Interestingly, under these conditions, the IFNα-induced expression of OAS2 (in hST2-U3C) and Ifit1 (in mST2-MS1KO) dramatically increased as compared with the control cells (Migr1-U3C and Migr1-MS1KO, respectively) (Figures 2C and 2D), with a maximum expression after 24 h of IFNα treatment. In contrast with the WT cells (Figures 1A and 1B), the expression of these genes in the human and mouse STAT1 KO cells overexpressing STAT2 was prolonged, which correlated with the continued presence of phosphorylated STAT2. Interestingly, knocking down STAT1 expression in MEF WT, resulted in a similar prolonged IFNα-induced expression pattern for Ifit1 and Oas2 as compared with control cells (data not shown). This implies that by increasing levels of STAT2 in STAT1 KO the IFNα response can be restored.

### STAT2 and IRF9 interact and mediate an IFNα response in the absence of STAT1

**Figure 3:**
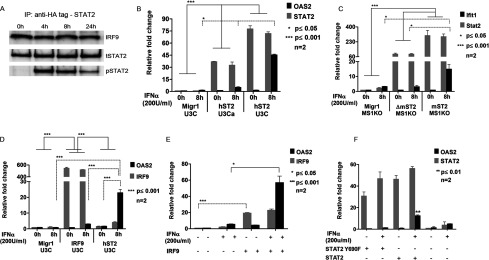
STAT2 and IRF9 complex and mediate an IFNα response in the absence of STAT1 (**A**) The interaction between STAT2 and IRF9 was analyzed by immunoprecipitation. hSTAT2-U3C were treated with IFNα for the indicated times. Cell lysates were immunoprecipitated with anti-HA antibody followed by Western blotting with IRF9, tSTAT2 and pSTAT2 antibodies. (**B**) Two different clones of hST2-U3C (hST2-U3Ca and hST2-U3C) varying in hSTAT2 expression level and their control Migr1-U3C; (**C**) ΔmST2-MS1KO, mST2-MS1KO and their control Migr1-MS1KO; (**D**) Migr1-U3C, IRF9-U3C and hST2-U3C; (**E**) hST2-U3C transiently transfected with Migr1-IRF9 (500 ng); (**F**) U3C cells transiently transfected with STAT2-Y690F or STAT2 plasmid (2.5 μg) were all treated with or without 200 U/ml IFNα for 8 h (**B**–**E**) or 24 h (**F**). Total RNA was extracted and OAS2, Ifit1, STAT2 or IRF9 relative fold inductions were quantified using qRT-PCR. Statistical significance is presented as compared with the non-treated control (results are means ± S.E.M.). Statistical analysis was conducted using one-way ANOVA with Tukey's *post hoc* test except in (**E**) where a Student's *t*-test, two-tailed, was used. *P≤0.05, **P≤0.01.

To prove that a STAT2/IRF9-containing complex is responsible for the IFNα response in the STAT1 KO cells overexpressing STAT2, we performed additional experiments. First, by using protein extracts from hST2-U3C cells treated with IFNα for increasing times in combination with anti-HA antibodies to immunoprecipitate STAT2, we were able to observe specific STAT2/IRF9 complex formation even after 24 h of IFNα treatment (Figure 3A; input control is shown in Figure 2A). Interestingly, the STAT2/IRF9 complex could already be detected in the absence of IFNα treatment (lane 1, Figure 3A), and was not affected by increased STAT2 phosphorylation. On the other hand, the phosphorylation kinetics of STAT2 correlated with the prolonged expression pattern of OAS2 (Figures 2A and 2C). We also checked the level of ISG expression in response to IFNα in two different clones of hST2-U3C with varying STAT2 mRNA levels. In hST2-U3C, the STAT2 mRNA level was 75-fold higher than in Migr1-U3C control, whereas in hST2-U3Ca there was a 30-fold difference (Figure 3B). This correlated with the difference in expression of OAS2 in these two cell lines in response to IFNα, being 9-fold higher in hST2-U3C (46-fold) as opposed to hST2-U3Ca (5-fold), when compared with untreated cells (Figure 3B). In addition to mST2-MS1KO cells, we generated a MS1KO stable cell line overexpressing a C-terminally truncated form of mSTAT2 (ΔmST2-MS1KO), which lacks the *trans*-activation domain of STAT2 and acts as a dominant negative. As shown in Figure 3(C), the levels of STAT2 in mST2-MS1KO cells correlated with the high induction of mouse Ifit1. ΔmST2-MS1KO facilitated no significant induction of the mouse Ifit1 gene in response to IFNα. Subsequently, we investigated in more detail the role of IRF9 in the IFNα response in the absence of STAT1. We generated a U3C cell line stably overexpressing IRF9 (IRF9-U3C). Interestingly, OAS2 expression increased only 3-fold as compared with Migr1-U3C cells after 8 h of IFNα treatment (Figure 3D). However, hST2-U3C cells transiently transfected with IRF9 showed a 10-fold increase in OAS2 gene expression in comparison with the hST2-U3C IFNα-treated cells and a 57-fold increase in contrast with Migr1-U3C cells (Figure 3E). Finally, we compared expression of IFIT2 and OAS2 in U3C cells transiently transfected with STAT2 or the tyrosine mutant STAT2Y690F (mutant form of STAT2 that cannot be phosphorylated on tyrosine). U3C-ST2 showed a 10-fold increase upon IFN treatment, whereas U3C-ST2Y690F exhibited no response, implying that the STAT2/IRF9-mediated IFNα-response is dependent on STAT2 phosphorylation. Together, these results point to the importance of the STAT2/IRF9 complex in the prolonged IFNα response in the absence of STAT1 and suggest an ISGF3-like function.

### STAT2/IRF9 and ISGF3 regulate expression of a common set of ISGs with different kinetics

**Figure 4:**
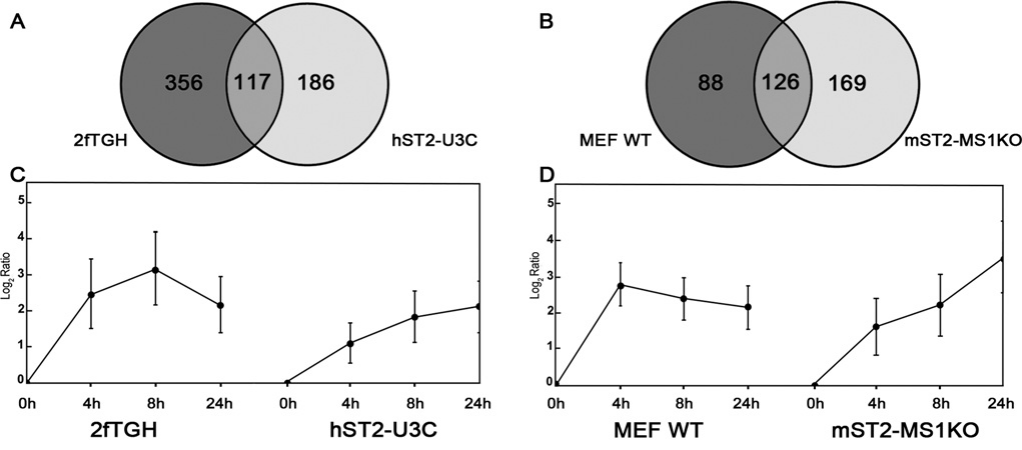
STAT2/IRF9 and ISGF3 regulate expression of a common set of ISGs with different kinetics (**A**) 2fTGH and hST2-U3C or (**B**) MEF WT and mST2-MS1KO were treated with IFNα for 0 h, 4 h, 8 h and 24 h and subjected to microarray analysis. Common up-regulated genes were selected by comparing transcriptomes of individual cell lines. Statistically significant up-regulated genes in human (**A**) and mouse (**B**) cell-line data sets were compared by Venn diagram analysis. Average expression profiles of common up-regulated genes between (**C**) 2fTGH and hST2-U3C and (**D**) MEF WT and mST2-MS1KO are displayed in centroid view. Expression values are shown as log_2_ ratio; error bars=S.D.

**Figure 5:**
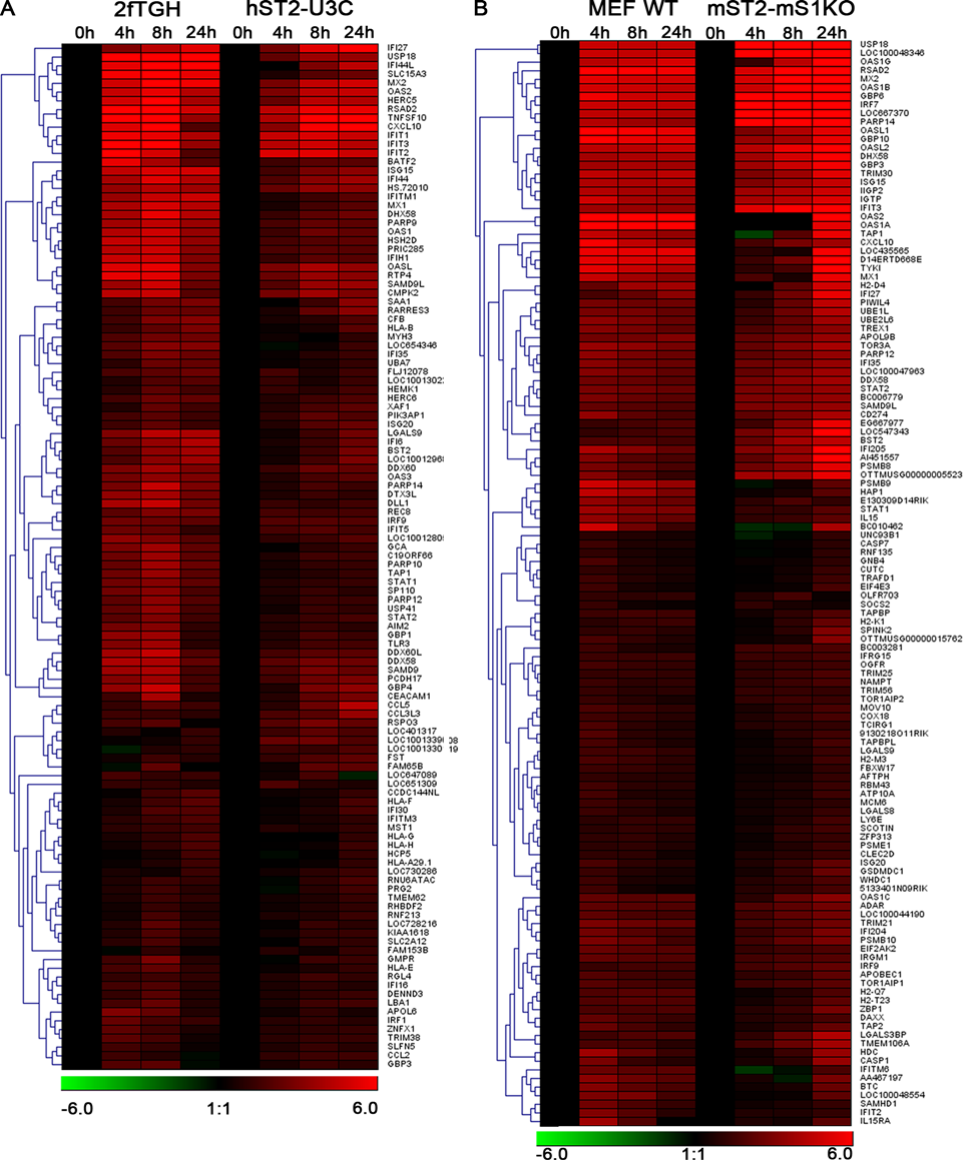
STAT2/IRF9- and ISGF3-mediated transcriptional responses predict functional overlap Cluster analysis of common up-regulated genes between (**A**) 2fTGH and hST2-U3C or (**B**) MEF WT and mST2-MS1KO. Total RNA from IFNα-treated cell lines was analyzed using Illumina Human HT-12 v4 (**A**) or MouseRef 8v2 (**B**) microarrays. For microarray analysis, background subtraction and quantile normalization were used, genes with ratio ≥2 and *P*≤0.05 were considered as up-regulated. log_2_ ratios from up-regulated genes were clustered using average linkage method.

To characterize IFNα-mediated transcriptional responses and identify the genes being regulated by STAT2/IRF9 in relation to ISGF3, we performed microarray experiments comparing human and mouse STAT1 KO cells overexpressing STAT2 with their WT counterparts treated with IFNα for 4 h, 8 h and 24 h. After quality check and data analysis, we only focused on the up-regulated genes. By comparing the expression profiles of hST2-U3C with 2fTGH, we identified 303 up-regulated genes in hST2-U3C of which 117 were in common with 2fTGH (Figure 4A). Similarly, by comparing the expression profiles of mST2-MS1KO with MEF-WT, we identified 295 up-regulated genes with 126 genes commonly induced between the two cell lines (Figure 4B). To characterize these commonly up-regulated genes in more detail, first we performed hierarchical cluster analysis (based on average linkage clustering of ratios) comparing human 2fTGH with hST2-U3C and mouse MEF-WT with mST2-MS1KO (Figures 5A and 5B, respectively). Strikingly, among the commonly induced genes in both human and mouse cell lines many known ISGs could be recognized, including IFITs, IFIs, ISGs, OASs, MX, radical S-adenosylmethionine domain-containing (RSAD2) and HECT and RLD domain-containing E3 ubiquitin protein ligase 5 (HERC5). In general, the induction level of these genes was lower in the STAT1 KO cells overexpressing STAT2 as opposed to WT cells. The centroid view, representing the average gene expression pattern in human (Figure 4C) and mouse (Figure 4D) cells unveiled a prolonged profile in hST2-U3C and MST2-MS1KO cells in response to IFNα. In contrast, in the WT cells, this was early and transient. In order to validate the microarray data, qRT-PCR was performed for a selection of these genes. Indeed, IFIT1, IFIT2, IFIT3, ISG15 and MX1 exhibited a prolonged IFNα-induced expression profile in hST2-U3C as compared with the 2fTGH cells (not shown). The same was true for the expression of Mx2, Ifit3, Isg15, Oas1b and RSAD2 when compared with MST2-MS1KO versus MEF-WT (not shown). Collectively, our results reveal that STAT2/IRF9 and ISGF3 regulate expression of a common set of ISGs, however, with a different kinetics.

### STAT2/IRF9 and ISGF3-mediated transcriptional responses predict functional overlap

**Figure 6:**
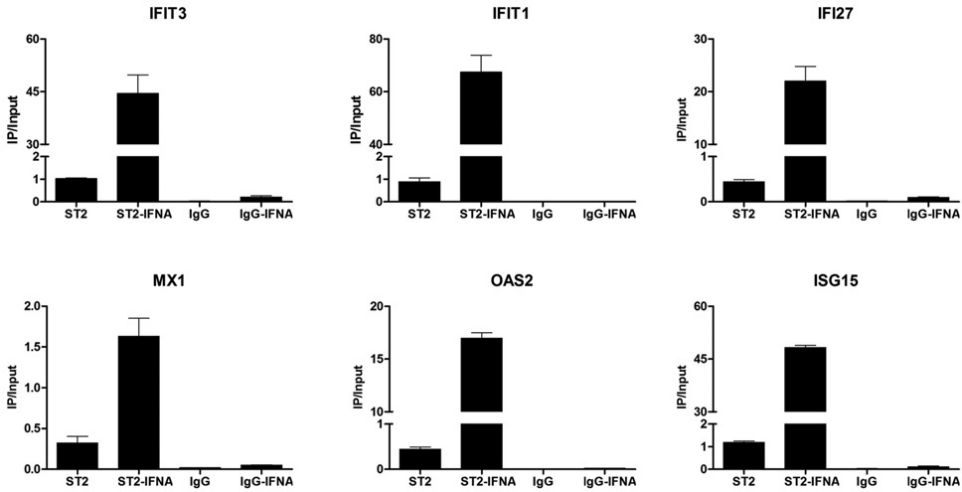
ChIP-qPCR analysis show enhanced binding of STAT2 to the ISRE of the IFI27, MX1, OAS2, IFIT1, IFIT3 and ISG15 genes in an IFNα-dependent manner in the hST2-U3C cells. Immunoprecipitated DNA was quantified by qPCR and normalized to values obtained after amplification of unprecipitated (input) DNA ChIP- qPCR confirms enhanced binding of STAT2 to the ISRE in an IFNα-dependent manner in the absence of STAT1.

Next, GO enrichment was performed on the commonly up-regulated genes in human and mouse WT and STAT1 KO cells overexpressing STAT2 (Table 1). Interestingly, based on the log_10_ *P*-value parameter, the categories that were highly overrepresented in both species displayed its main involvement in three groups: (1) ‘response to virus’ (white) including GO categories such as defence response or regulation of viral reproduction; (2) ‘response to stimulus’ (light grey) including response to cytokine or biotic stimulus categories; and (3) ‘multi-organism processes’ (dark grey) including response to stress and organic substance. We subsequently examined the top-20 commonly up-regulated genes in 2fTGH versus hST2-U3C derived from the ‘response to the virus’ category based on the 24 h expression profile of hST2-U3C. Indeed, these genes included well known ISGs with antiviral functions such as IFIT1, IFIT2, IFIT3, interferon alpha-inducible protein (IFI)27, IFI44, IFI44L, OAS1, OAS2, OASL, ISG15, MX1 and RSAD2 (Table 2). Using Pscan we confirmed the presence of a classical ISRE in the promoter of all of these genes (Table 2). BioMart from Ensemble successively allowed us to identify mouse homologues for these 20 human genes. For six of these genes we found more than one mouse homologue, including OASL and IFI27 (Table 2, indicated by **), whereas no mouse homologue was identified for HERC5. Ifit27l1, Ifi44l and DEAD (Asp-Glu-Ala-Asp) box polypeptide 60 (Ddx60) mouse gene probes were not present on the mouse beadchip array (Table 2, indicated by *). On the other hand, the probe for mouse Ifit1 failed on the array (Table 2, indicated by ***) although our qPCR experiments showed comparable results to human IFIT1 (data not shown). All of the identified mouse homologues also contained a classical ISRE sequence in their promoter, which correlated with a similar expression pattern as compared with their human equivalents (Table 2). Performing ChIP-qPCR on hST2-U3C treated with or without IFNα and using antibodies against STAT2 or IgG clearly showed enhanced binding of STAT2 to the ISRE of the IFI27, MX1, OAS2, IFIT1, IFIT3 and ISG15 genes, in an IFNα-dependent manner (Figure 6). Together with the cluster analysis, this strongly implied functional overlap between STAT2/IRF9 and ISGF3 in human and mouse cells, especially for the potential of generating an IFNα-induced antiviral response.

**Table 1:** Gene ontology enrichment Common up-regulated genes from human and mouse microarray experiments were taken for gene ontology enrichment analysis using Gorilla and Revigo software. Gene ontology terms were grouped as follow: top eight terms classified as ‘response to virus’ (white background), next five were categorized as ‘response to stimulus’ (light grey background) and last six as ‘multi-organism processes’ (dark-grey background) based on the log_10_ *P*-values. Frequency scores were the percentage of proteins in UniProt which were annotated with a GO term in the GOA database, i.e. a higher frequency denotes a more general term.

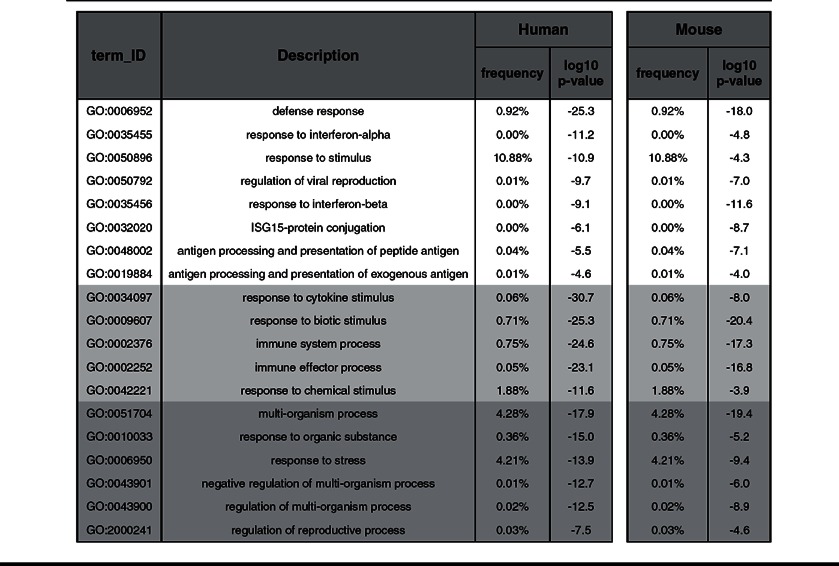

**Table 2:** The top-20 commonly up-regulated antiviral genes in 2fTGH versus hST2-U3C Cells were untreated or stimulated with IFNα for 4 h, 8 h and 24 h. Expression ratios (of treated versus untreated control) were calculated as means from three (human) and two (mouse) repeats. Genes were selected from the ‘response to the virus’ GO category (Table 1). Mouse homologues (indicated as the percentage homology with the human gene) were identified using Ensemble BioMart. P: position of the first nucleotide in the predicted ISRE sequence in relation to the transcriptional start site. S: consensus ISRE matching score (from 0 to 1), with 1 representing 100% identity.

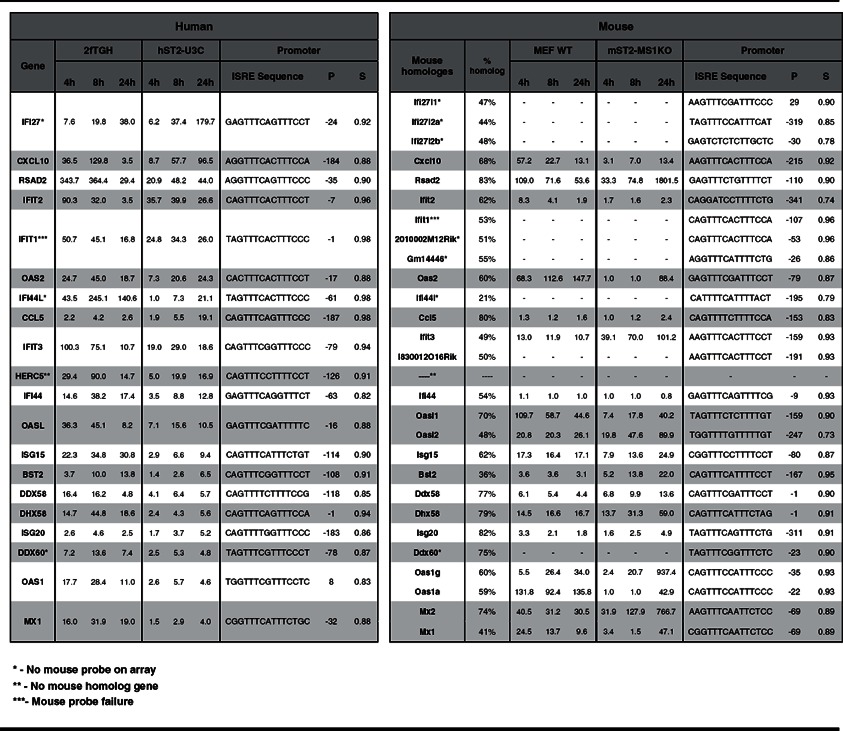

### STAT2/IRF9 regulates expression of ISRE-independent ISGs

**Figure 7:**
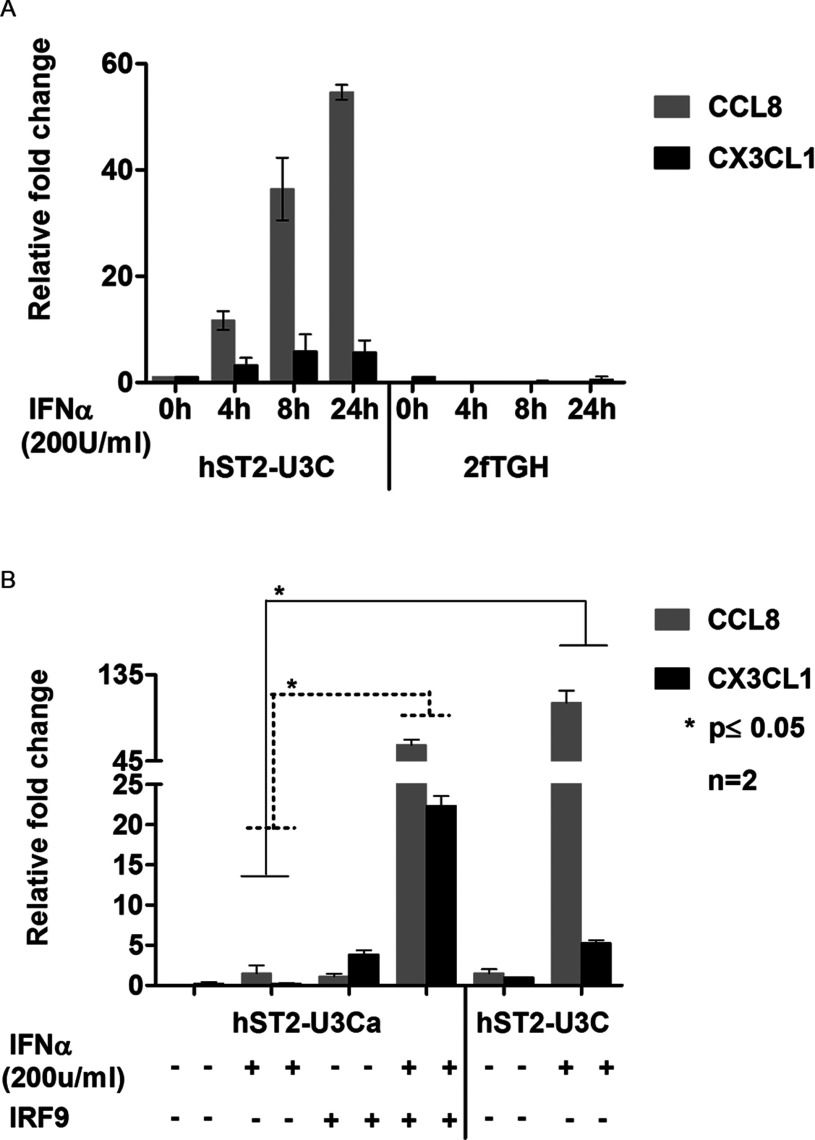
STAT2/IRF9 regulates expression of ISGF3-independent genes (**A**) 2fTGH and hST2-U3C were treated with IFNα for indicated times. (**B**) Two different clones of hST2-U3C (hST2-U3Ca and hST2-U3C) varying in hSTAT2 expression levels were treated with IFNα for 8 h. Subsequently, hST2-U3Ca was transfected with Migr1-IRF9 (500 ng) and treated with IFNα for 8 h. In (**A**, **B**), total RNA was extracted. CCL8 and CX3CL1 relative fold induction was quantified using qRT-PCR. All data are presented as means±S.E.M. Statistical significance was assessed using Student's *t*-test, two tailed, *P≤0.05.

Comparing the expression profiles of hST2-U3C with 2fTGH also identified 186 genes specifically up-regulated in hST2-U3C cells (Figure 4A). Table 3 illustrates the top ten of these genes, of which the expression of chemokine (C-C motif) ligand 8 (CCL8) and chemokine (C-X3-C motif) ligand 1 (CX3CL1) was confirmed by qRT-PCR in hST2-U3C and 2fTGH after IFNα treatment (Figure 7). As shown in Figure 7(A), the expression of CCL8 and CX3CL1 depended on both STAT2 and IRF9, but was absent from WT cells. Indeed, their IFNα-induced expression correlated with the STAT2 levels in hST2-U3C and hST2-U3Ca. Moreover, hST2-U3C cells transiently transfected with IRF9 showed increased expression of CCL8 and CX3CL1 in comparison with the hST2-U3C in response to IFNα (Figure 7B). Detailed promoter analysis of these genes did not identify a classical ISRE motif, implying a different mode of regulation. This suggests that STAT2/IRF9 also regulates expression of ISRE-independent ISGs.

**Table 3:** The top ten STAT1/IRF9-specific genes regulated in response to IFNα hST2-U3C cells were untreated or stimulated with IFNα for 4 h, 8 h or 24 h. Total RNA from each sample was analyzed using Illumina Human HT-12 v4 microarrays. Expression ratios (of treated versus untreated control) were calculated as the averages from three repeats.

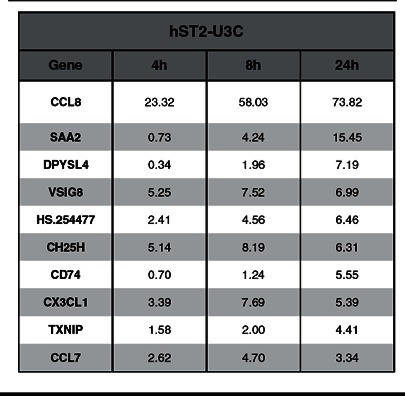

### STAT2/IRF9 mediates a similar antiviral response against EMCV and VSV virus as ISGF3

**Figure 8:**
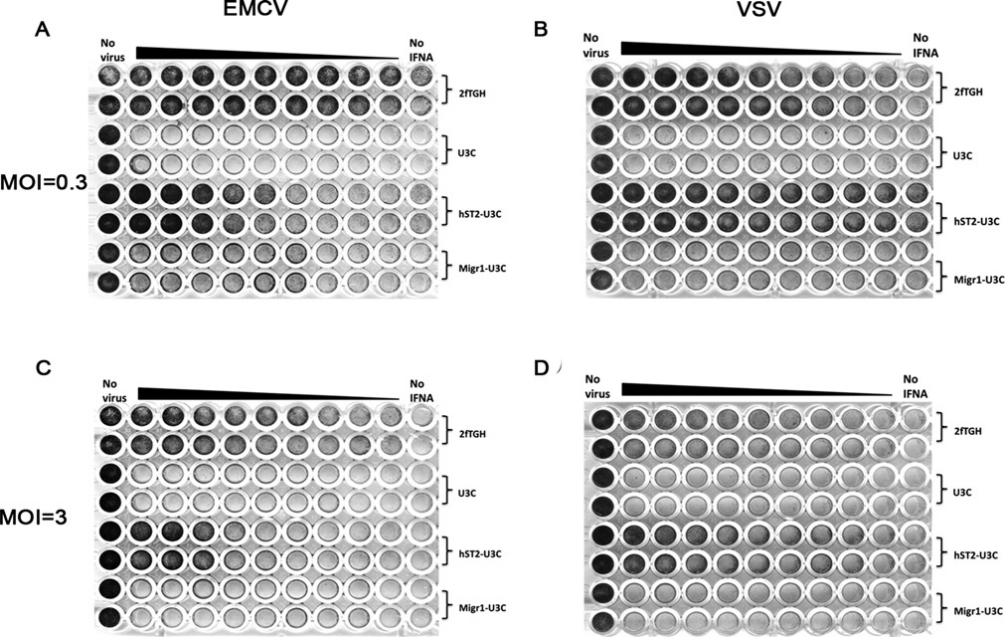
2fTGH, U3C, hST2-U3C and Migr1-U3C cell lines, pre-treated for 24 h with 2-fold serial dilutions of IFNα from 250 U/ml, were infected with (**A**) EMCV or (**B**) VSV at a MOI of 0.3 for 20 h, or at a MOI of 3 for 20 h (**C** and **D**, respectively) followed by visualizing live cells by crystal violet staining STAT2/IRF9 mediates a similar antiviral response against EMCV and VSV as ISGF3.

To provide further evidence for the functional overlap between STAT2/IRF9 and ISGF3 in the antiviral response, we performed a series of antiviral assays on 2fTGH, U3C, hST2-U3C and Migr1-U3C cells (Figure 8). The cells were first pretreated with 2-fold serial dilutions of IFNα for 24 h and subsequently infected with either EMCV or VSV with MOI=0.3 (Figures 8A and 8B) or 3 (Figures 8C and 8D) for each virus. Indeed, we could observe a restored antiviral response in hST2-U3C cells, as compared with 2fTGH, due to the overexpression of STAT2. U3C cells showed no antiviral protection as well as the Migr1-U3C control cells even when treated with the lower virus concentration of MOI=0.3. In conclusion, STAT2/IRF9 mediates a similar antiviral response against EMCV and VSV virus as ISGF3.

## DISCUSSION

Previously, we showed that STAT2 homodimers interact with IRF9 (STAT2/IRF9) to activate transcription of ISRE containing ISGs in response to IFNα [7]. Indeed, evidence is accumulating for the existence of a STAT1-independent IFNα signalling pathway, where STAT2/IRF9 can substitute for the role of ISGF3 [14–16]. Here, we provide further insight into the genome-wide transcriptional regulation and the biological implications of STAT2/IRF9-dependent IFNα signalling as compared with ISGF3.

By comparing the timely IFNα response of human and mouse WT cells, we observed an early and transient character that correlated with the phosphorylation kinetics of the ISGF3 components STAT1 and STAT2 and the presence of IRF9 (Figure 1). The expression of the classical ISGs OAS2 and Ifit1 followed this pattern, confirming the transient ISGF3-dependent IFNα-response displayed in many different cell types [6]. As expected, in STAT1 KO cells this ISGF3-dependent IFNα-response was severely abrogated, highlighting the importance of STAT1 [32]. However, IFNα-induced STAT2 phosphorylation was also diminished in these cells, which associated with decreased but still detectable expression levels of OAS2 and Ifit1.

Interestingly, increasing the levels of STAT2 in the human and mouse STAT1 KO cells recapitulated the IFNα response. In contrast with the WT cells (Figure 1), the IFNα-induced expression of OAS2 and Ifit1 in the human and mouse STAT1 KO cells overexpressing STAT2 was prolonged, which correlated with the kinetics of STAT2 phosphorylation, and the presence of a STAT2/IRF9 complex requiring STAT2 phosphorylation and the STAT2 transactivation domain (Figures 2 and 3). This response also depended on the levels of IRF9, as transient overexpression of IRF9 in hST2-U3C cells further increased the response of OAS2 to IFNα (Figure 3). On the other hand, U3C cells overexpressing IRF9 only weakly responded to IFNα, in agreement with the lack of intrinsic transcriptional capacity of IRF9 [33] and limited amount of phosphorylated STAT2 in these cells after treatment (not shown). A similar prolonged IFNα-induced expression pattern for Ifit1 and Oas2 could be detected after knocking down STAT1 expression in MEF WT (data not shown), suggesting that activation of STAT2/IRF9-dependent transcription depends on the level of STAT1 in WT cells.

Our findings are in agreement with Lou et al. [12], who observed that the STAT2/IRF9 complex effectively drives transcription of the RIG-G gene in U3A cells upon IFNα treatment, in a STAT1-independent manner. U3A and U3C cells belong to the same complementation group of IFNα- and IFNγ-unresponsive mutants [17]. However, in U3A cells RIG-G expression required overexpression of both STAT2 and IRF9 [12], whereas in our study in U3C cells overexpression of STAT2 was sufficient. Lou et al. [12] also showed IFNα-independent interaction of STAT2 and IRF9, but transcriptional regulation of RIG-G required STAT2 phosphorylation (not shown). Further comparison of U3A and U3C learned that IFNα-induced STAT2 phosphorylation in U3A cells is also severely diminished (although still visible) as compared with 2fTGH WT cells (not shown). However, IFNα-induced expression of OAS2 is not detectable (not shown). IRF9 levels, on the other hand, were lower in U3A as compared with U3C (not shown), and only up-regulated by IFNα in U3C and not in U3A (not shown). Together this implies that, in the absence of STAT1, a certain threshold amount of STAT2 and IRF9 must be reached to allow STAT2 phosphorylation and STAT2/IRF9-mediated transcription. Subtle differences between U3C and U3A in these threshold levels could potentially explain for the differences in their response to IFNα. Our experiments in MS1KO fibroblasts, in which the presence of IFNα-induced STAT2 phosphorylation and IRF9 correlate with significant induction of ISG transcription, are in agreement with this.

Likewise, Bowick et al. [34] showed a prolonged IFNα response of STAT1 KO mice to viral infection, resulting in prolonged expression of classical ISGs. Perry et al. on the other hand, confirmed the association of STAT2 with the promoter of antiviral genes induced in response to Dengue virus in STAT1-deficient mice [11]. Similarly, Kraus et al. [33] and Poat et al. [35], observed that a hybrid of IRF9 and STAT2 recapitulates interferon-stimulated gene expression in the absence of STAT1. We extend these observations by showing that abundance of phosphorylated STAT2 and IRF9 allows a STAT2/IRF9 complex to regulate transcription of ISGs, resulting in a prolonged expression pattern, in both human and mouse cells independent of STAT1.

Remarkably, the STAT2/IRF9 complex formed in the STAT1 KO cells overexpressing STAT2 could already be detected in the absence of IFNα treatment (Figure 3), suggesting that the interaction was independent of STAT2 phosphorylation. This could also suggest that STAT2 phosphorylation takes place while complexed with IRF9. Our results are in disagreement with the model proposed by Tang et al. [36], in which STAT2/IRF9 complex formation depends on IFNα-induced acetylation. However, this model is not compatible with the frequently made observations that histone deacetylase inhibitors (HDACi) block IFN signalling [37]. On the other hand, others have shown that STAT2 and IRF9 interact independently of phosphorylation [12] and that nucleo-cytoplasmic shuttling of STAT2 has been attributed to the constitutive binding of STAT2 to the nuclear localization signal (NLS)-containing IRF9, independent of phosphorylation, to transport STAT2 into the nucleus [38]. In agreement with Testoni et al. who used ChIP-chip with anti-STAT2 antibodies, a substantial percentage of ISG promoters have shown to be occupied by un-phosphorylated STAT2 before IFNα treatment [39]. On the other hand, IFNα treatment of hST2-U3C cells stimulated the interaction of phosphorylated STAT2 with IRF9, even after 24 h, which closely correlated with the prolonged expression pattern of OAS2 (Figures 2 and 3). This was again in accordance with Testoni et al., who observed that the majority of promoters that gained STAT2 in response to IFNα was positive for phosphorylated STAT2 [39] and therefore predicted that the STAT2/IRF9 complex functioned similar to the classical ISGF3-directed pathway.

Subsequent microarray analysis of IFNα-treated human and mouse WT and STAT1 KO cells overexpressing STAT2 extended our initial observations and identified ∼120 known ISRE-containing ISGs commonly up-regulated by STAT2/IRF9 and ISGF3 (Figures 4A and 4B). The STAT2/IRF9-directed expression profile of these ISGs was prolonged as compared with the early and transient response mediated by ISGF3, implying that STAT2/IRF9 and ISGF3 regulate expression of a common set of ISGs with different kinetics. In general, in WT cells, the transient nature of the IFNα response is tightly regulated by up-regulation of suppressor of cytokine signalling 1 (SOCS1) [40]. In contrast, IFNα treatment of hST2-U3C and mSTAT2-MS1KO cells did not result in increased expression of SOCS1 (data not shown), which could explain the prolonged phosphorylation kinetics of STAT2 and expression pattern of ISGs.

Among the commonly induced genes in both human and mouse cell lines were many known ISGs (Figure 5), and functional analysis revealed significant enrichment in biological functions categorized in ‘response to virus’ (defence response, regulation of viral reproduction), ‘response to stimulus’ (response to cytokine or biotic stimulus) and ‘multi-organism processes’ (response to stress and organic substance) (Table 1). Interestingly, the top-20 commonly up-regulated human genes from the ‘response to virus’ category, with their mouse homologues, predominantly consisted of well characterized ISGs with known antiviral functions (Table 2). Within the promoter of all of these genes, we confirmed the presence of a classical ISGF3-binding ISRE. Indeed, ChIP-qPCR confirmed binding of STAT2 to a selection of these genes, in an IFNα-dependent manner in the absence of STAT1 (Figure 6). This strongly implies functional overlap between STAT2/IRF9 and ISGF3 in human and mouse cells, especially for the potential of generating an IFNα-induced antiviral response. Indeed, hST2-U3C cells were able to trigger the antiviral response upon EMCV and VSV infection, protecting better against VSV as compared with EMCV (Figure 8), offering additional proof for the functional overlap between STAT2/IRF9 and ISGF3. Thus, STAT2/IRF9 not only activates expression of known antiviral ISGs, but also has a biological function in the reconstitution of the antiviral response in cells lacking STAT1. This is in agreement with findings of Kraus et al. [33] and Poat et al. [35], who observed that expression of the IRF9/STAT2 fusion can recapitulate the Type I IFN biological response, producing a cellular antiviral state that protects cells from RNA and DNA virus-induced cytopathic effects and inhibits virus replication.

Previously, Sarkis et al. [10] proposed a novel STAT1-independent IFNα signalling pathway in human liver cells that depended on STAT2 and IRF9. IFNα induction of the antiviral protein A3G and other ISGs [protein kinase, interferon-inducible double-stranded RNA-dependent activator (PKR), ISG15 and myxovirus (influenza virus) resistance 1 (MX1)] was STAT1-independent, but STAT2-dependent, in these cells. Similarly, Lou et al. [12] showed that the STAT2/IRF9 complex effectively drives transcription of the RIG-G (IFIT-3) gene in NB4 cells upon signalling cross-talk between retinoic acid and IFNα, in a STAT1-independent manner. Moreover, it was shown that the late antiviral gene DUOX2 was induced by an autocrine/paracrine pathway specifically triggered in airway epithelial cells by synergistic action of IFNβ and TNFα, and depending on STAT2/IRF9 but entirely independent of STAT1 [13]. Of these genes, IFIT3, PKR, ISG15 and MX1 were both regulated by STAT2/IRF9 and ISGF3 in our human and mouse cell lines. However, A3G was only regulated by ISGF3 and not by STAT2/IRF9. The expression of DUOX2 could not be detected in our WT and STAT2-overexpressing STAT1 KO cells, in response to IFNα, which could point to a cell-type-specific mechanism. Interestingly, Cheon et al. [41] recently identified another alternative, unphosphorylated (U)-ISGF3-mediated, IFNβ response pathway, which was shown to regulate a group of classical antiviral ISRE-containing ISGs but to act independently of STAT phosphorylation. This is in contrast with our results, in which un-phosphorylated STAT2 formed a complex with IRF9, but did not induce ISG expression in human and mouse cell lines (Figures 3B–3E), whereas STAT2 mutation (Y690F) impaired the ability to induce gene expression (Figure 3F). Therefore, STAT/IRF9-directed gene expression is clearly dependent on STAT2 phosphorylation.

The ISGF3 complex, consisting of STAT1–STAT2 heterodimers and IRF9, binds a composite DNA sequence (AGTTTCNNTTTCN) in which IRF9 contributes most of the DNA-binding specificity by recognizing the core sequence of the ISRE [42]. STAT1 contributes necessary contacts with DNA which raises the affinity of ISGF3 for DNA above a minimal threshold provided by IRF9 alone. STAT2 contains a transactivation domain that is essential for transcriptional activity of ISGF3 [43]. In the STAT2/IRF9 complex, STAT2 homodimers in conjunction with IRF9 recognize only a core ISRE sequence, resulting in a lower DNA-binding affinity as compared with ISGF3 [7]. The presence of classical ISGF3-binding ISRE sequences, also bound by STAT2/IRF9, in the promoters of the commonly induced ISGs in both human and mouse cell lines, thus could explain the functional overlap of STAT2/IRF9 with ISGF3. The lower DNA-affinity of the STAT2/IRF9 complex as compared with ISGF3, on the other hand, requires abundance of STAT2 and IRF9 protein and correlates with the delayed and prolonged nature of its IFNα-mediated activity. In addition, in the hST2-U3C cells we identified a group of ISGs, including CCL8 and CX3CL1, whose response to IFNα was absent from 2fTHG cells (Table 3). Moreover, the IFNα-induced expression of these genes depended on both STAT2 and IRF9 and were therefore classified as ‘STAT2/IRF9-specific’ (Figure 7). Detailed promoter analysis of the top-ten ‘STAT2/IRF9-specific’ genes did not identify a classical ISGF3-binding ISRE, predicting that a DNA sequence distinct from the ISRE is involved in the regulation of these ‘STAT2/IRF9-specific’ genes. Future ChIP-seq experiments will hopefully reveal the identity of this mechanism.

In analogy to the previously identified role of STAT2/IRF9 in the delayed transcriptional regulation of the RIG-G and DUOX2 genes, which correlated with prolonged STAT2 phosphorylation and STAT2 and IRF9 expression in a cell-type-specific manner, we hypothesize that STAT2/IRF9 can coexist with the classical ISGF3 complex only in cells with elevated levels of STAT2 and prolonged STAT2 phosphorylation. In contrast, in cell types with a transient STAT1 and STAT2 phosphorylation pattern, like 2fTGH, ISGF3 is the pre-dominant mediator of IFNα signalling. This situation is very likely to be cell-type-specific, where both complexes may be involved in different stages of the antiviral response; ISGF3 stimulating a rapid and transient antiviral response and STAT2/IRF9 being responsible for a more prolonged antiviral response. It also becomes clear that equal to phosphorylation, IFN signalling is regulated by acetylation. In particular, inhibition of STAT1 (but not STAT2) by acetylation has been observed in many systems [37] leading to termination of IFN signalling. Therefore, the presence of acetylated STAT1 in the ISGF3 complex (which can be achieved by HDACi or IFN pre-stimulation) seems incompatible with prolonged IFNα-dependent transcription. As STAT1 acetylation does not affect STAT2, it could be that under certain conditions STAT2/IRF9 may allow continuation of the IFNα response and prolonged transcription. This could provide a level of redundancy to certain cells to ensure effective induction of an antiviral state and help to overcome countermeasures that many viruses have evolved against IFN-dependent signalling, for example blocking STAT1 to impair the formation of ISGF3. Identifying these cell types and the role of STAT2/IRF9 in the regulation of specific transcriptional programmes and antiviral activity, as compared with ISGF3, is among our next challenges.
